# Supplementary figures and images for: Helminth infection driven gastrointestinal hypermotility is independent of eosinophils and mediated by alterations in smooth muscle instead of enteric neurons
Source: PLoS Pathog. 2024 Aug 14;20(8):e1011766. doi: 10.1371/journal.ppat.1011766 (PMC11346963; doi:10.1371/journal.ppat.1011766)

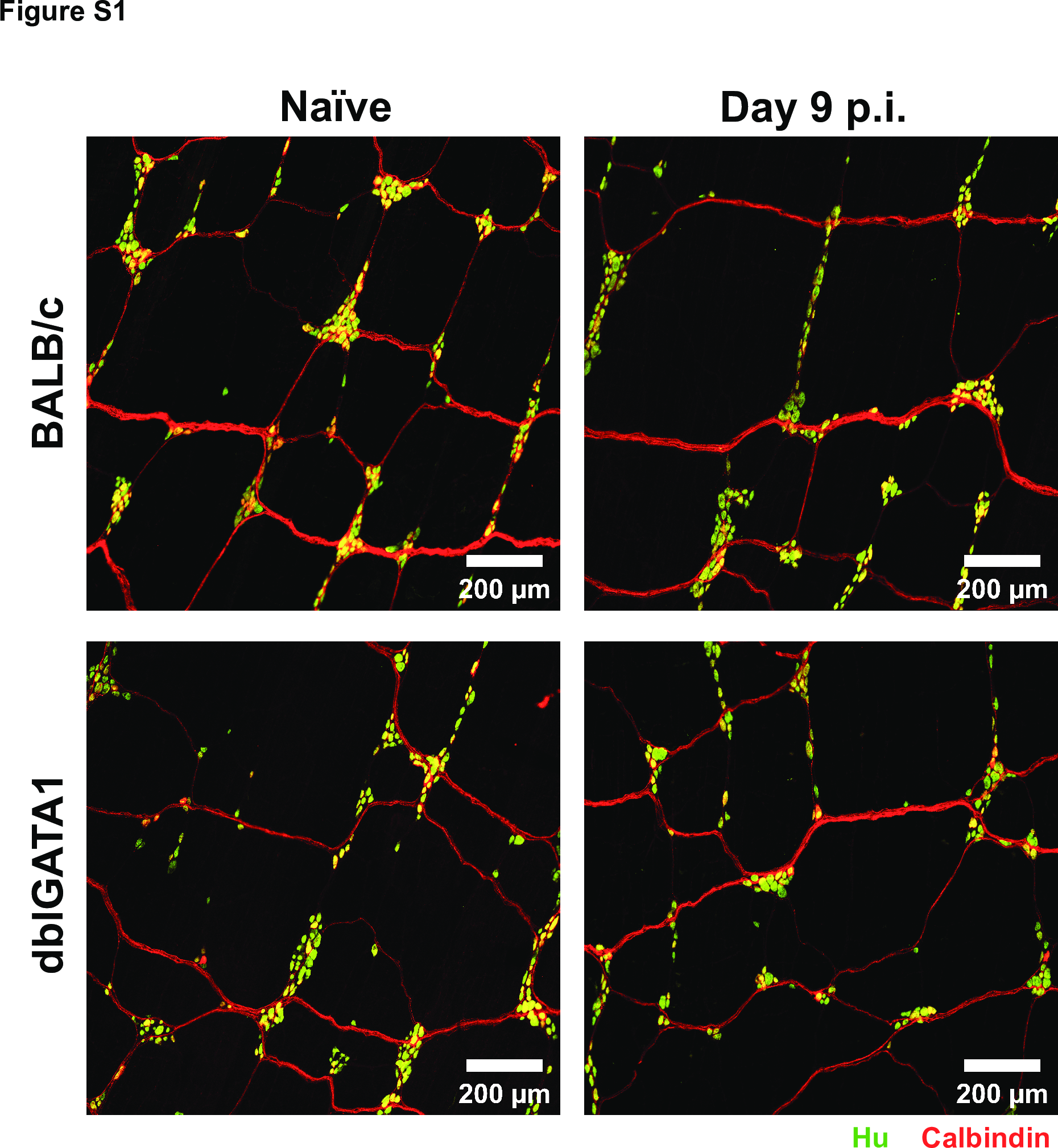

Supplement: S1 Fig — dblGATA1 mice and wildtype BALB/c controls were infected with 400–500 L3 Nb and sacrificed day 9 p.i. Control groups of naïve mice were included. Myenteric plexus layers were collected as detailed in Materials and Methods and stained with antibodies against the pan neuronal marker HuC/D (green) and calbindin (red). Representative images are shown from a single animal from each group. Intra-ganglionic connections were indicated as calbindin+ axonal bundles between ganglions. (TIF) [file ppat.1011766.s001.tif]

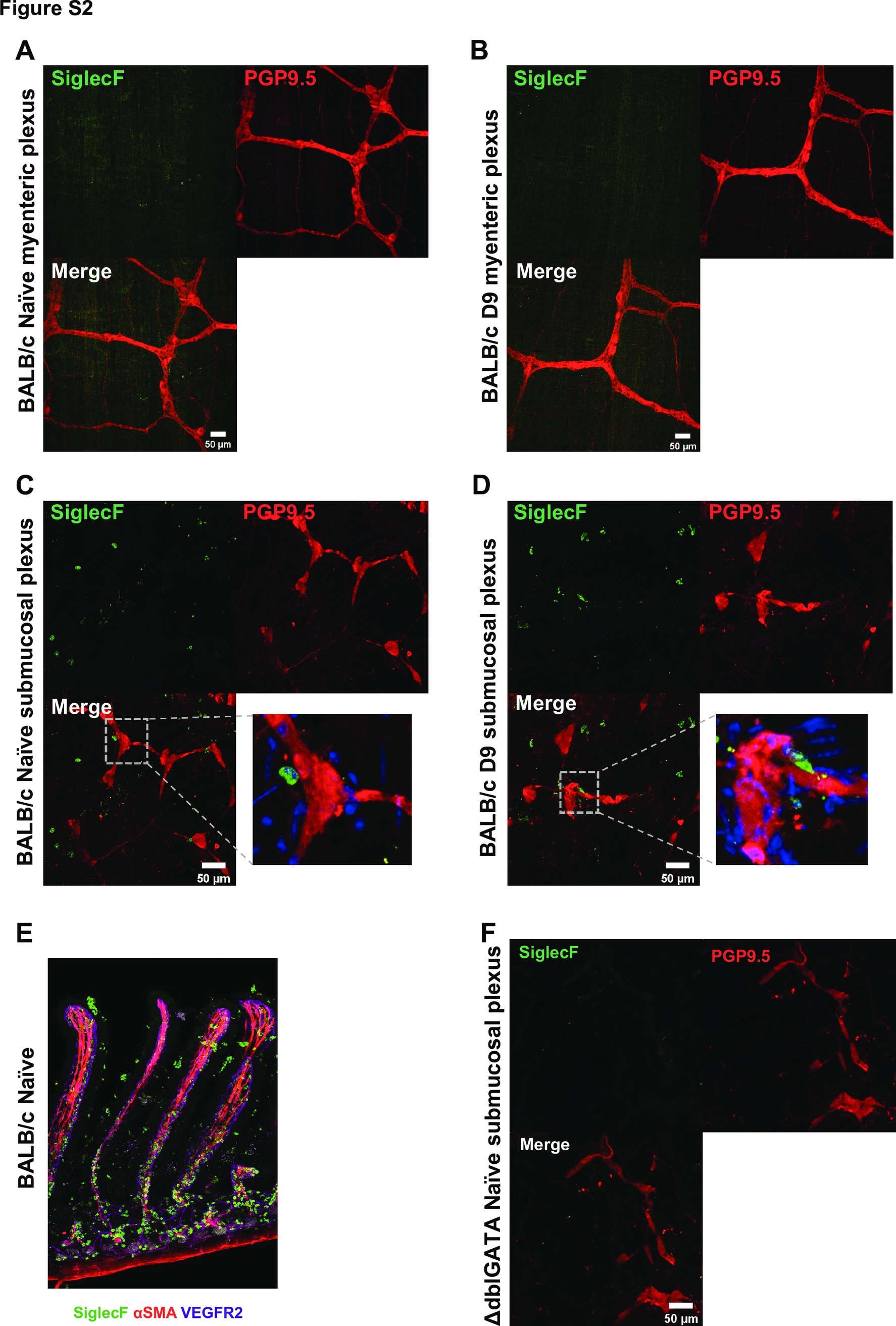

Supplement: S2 Fig — dblGATA1 mice and wildtype BALB/c controls were infected with 400–500 L3 Nb and sacrificed day 9 p.i. Control groups of naïve mice were included. Myenteric and submucosal plexus layers were collected as detailed in Materials and Methods and stained with antibodies against the pan neuronal marker PGP9.5 (red) and SiglecF (green). Representative images are shown for (A&B) the myenteric plexus and (C&D) the submucosal plexus of naïve and Nb infected BALB/c mice. Inserts in C&D show magnified areas were SiglecF+ eosinophils can be found in close proximity (<1 μm) with PGP9.5+ submucosal neurons. (E) Villous wholemounts from naïve BALB/c mice stained with antibodies against SiglecF (green), αSMA (red) and VEGFR2 (magenta). (F) Representative images of the submucosal plexus from a naive dblGATA1 mouse stained with antibodies against SiglecF (green) and PGP9.5 (red). (TIF) [file ppat.1011766.s002.tif]

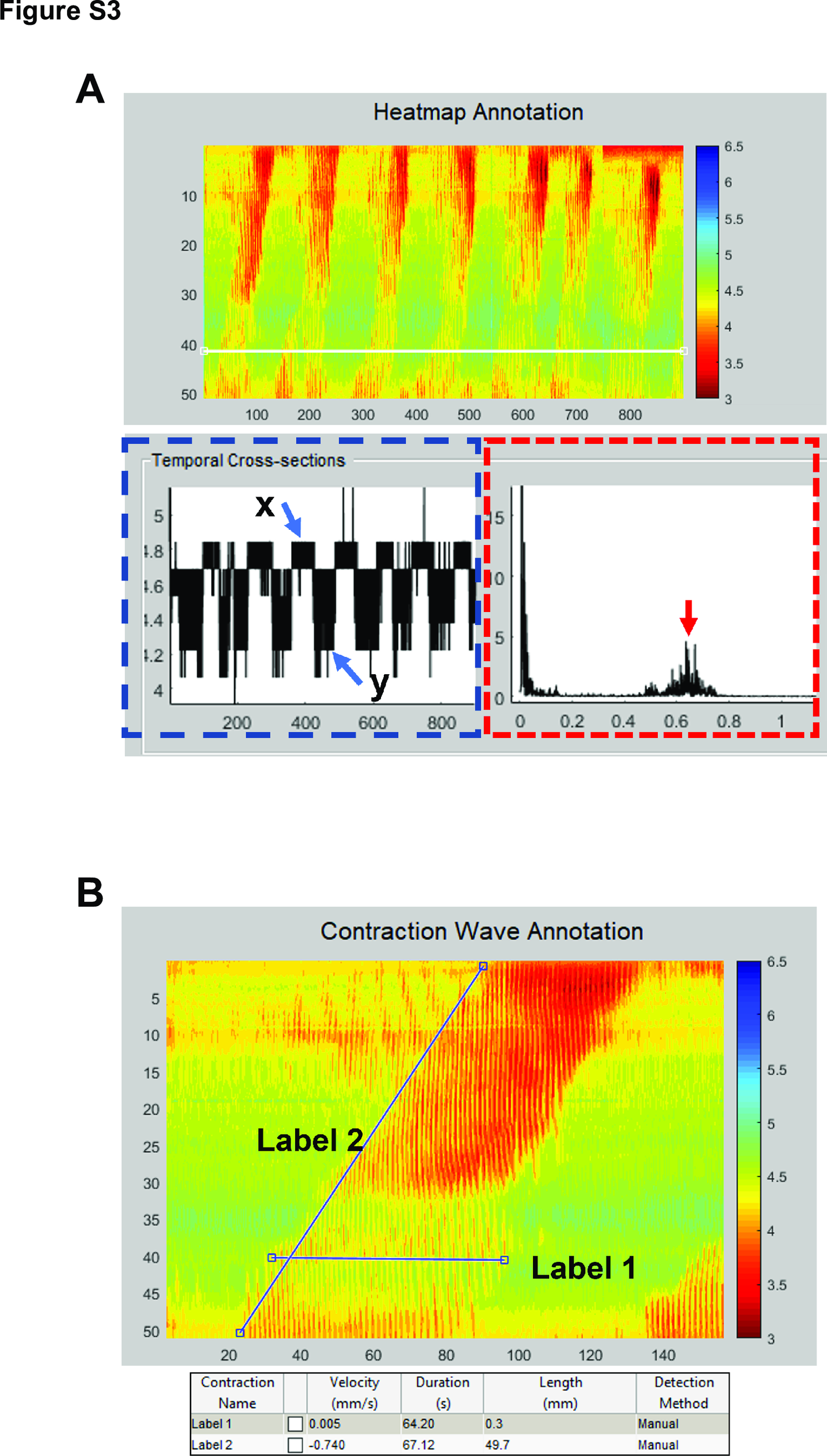

Supplement: S3 Fig — Results from video imaging experiments were analysed using Analyse 2 in MATLAB. The x-axis in the heatmap indicates time and y-axis indicates position of the tissue. Each colour coded pixel indicates the diameter of the jejunum at a given time and location. Neurogenic contractions are indicated by the warm coloured bands in the heatmap. To generate contraction amplitude and slow wave data, a horizontal line was drawn at the distal ¼ of the tissue using the Heatmap Annotation function (A). The software calculates the changes in tissue diameter (indicated by colour) at any time at the line location. Contraction amplitudes (A, blue box) were recorded as % changes in tissue diameter, which is calculated by dividing changes in diameter between relaxation (point x) and contraction (point y) by relaxation, or (x–y)/x. Slow wave information (A, red box) was performed using fast Fourier transformation that separates the high frequency slow wave contraction (red circle and arrow) from the low frequency neurogenic contractions. Contraction velocity and duration were analysed with the Contraction Wave Annotation function (B, zoomed in view of a contraction wave). Contraction duration was defined as how long a PCC contraction lasts within the distal ¼ of the tissue, as indicated by Label 1. Contraction velocity is defined as the absolute value of speed of a PCC contraction that travels from the distal end to the proximal end of the tissue (Label 2). (TIF) [file ppat.1011766.s003.tif]

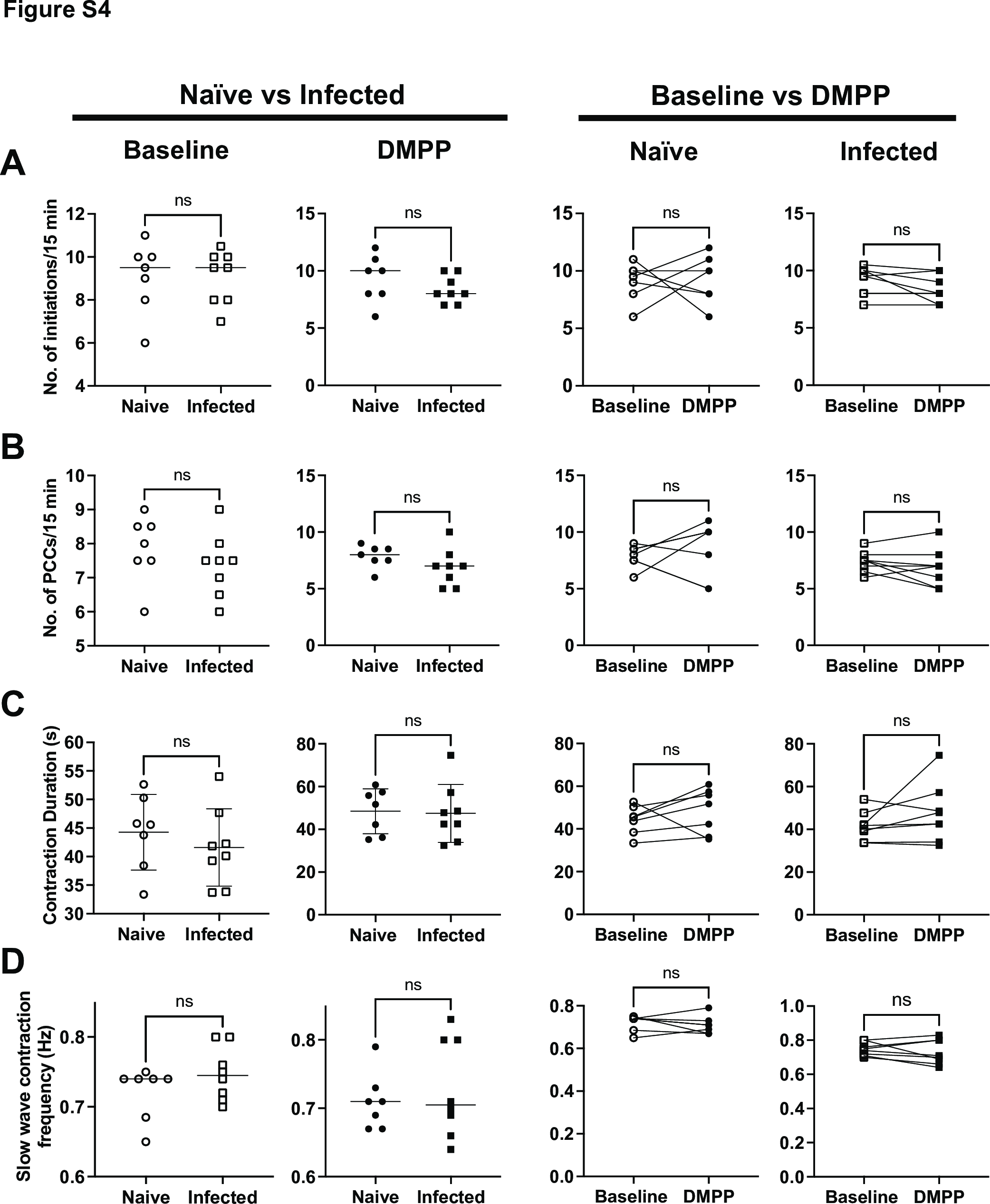

Supplement: S4 Fig — BALB/c mice were infected with 400–500 L3 Nb and sacrificed day 6–9 p.i. Control groups of naïve mice were included. Jejunal contractile activities were recorded under physiological conditions in a horizontal organ bath using a video camera, and data analysed with in-house software Scribble 2.0 and Analyse 2. Analysis of the spatiotemporal heatmap provided information on PCC contraction duration and velocity, contraction amplitude, and slow wave frequencies. Comparison of (A) neurogenic contraction initiations, (B) peristaltic contraction complex (PCC) frequencies, (C) neurogenic contraction duration and (D) slow wave contraction frequencies recorded in tissues from naïve or infected mice under baseline conditions or after DMPP application. Open and closed symbols indicate recordings taken during baseline conditions (open) or after DMPP application (closed). Symbols represent individual animals and individual experiments (n = 7–8 per group) with each experiment including tissues from one naive animal and one infected animal kept within the individual chambers of the same organ bath. Tissues that cease contracting before the end of each experiment were excluded from analyses. Data are shown as mean ± SEM. For (A&B) significance was determined by Mann-Whitney test when comparing naïve and infected mice and Wilcoxon signed-rank test when comparing baseline and DMPP. For (C&D), significance was determined using either unpaired (for naïve vs infected) or paired (baseline vs DMPP) student T-test. (TIF) [file ppat.1011766.s004.tif]

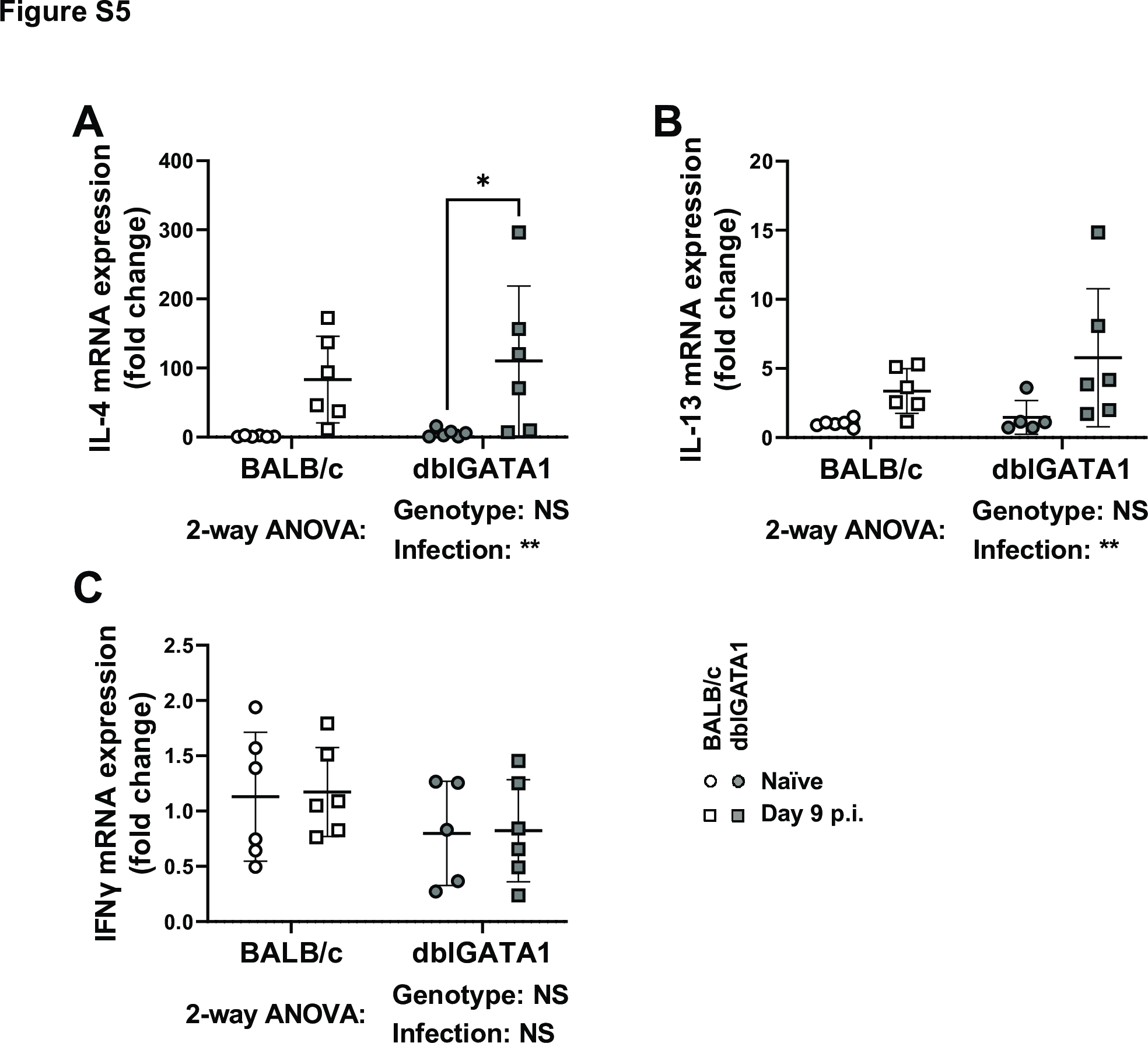

Supplement: S5 Fig — dblGATA1 mice and wildtype BALB/c controls were infected with 400–500 L3 Nb and sacrificed at day 9 p.i. Control groups of naïve mice were included. The expression of (A) IL4, (B) IL13 and (C) IFNγ were determined by qRT-PCR for whole jejunum tissue lysate. mRNA expressions in each animal were calculated using the Pfaffl method and presented as fold changes to the average of the housekeeping gene GAPDH. Symbols represent individual animals and data are shown as mean ± SEM from one experiment with n = 4 per group. The significance was calculated by two-way ANOVA with Tukey’s post-hoc analysis. (TIF) [file ppat.1011766.s005.tif]

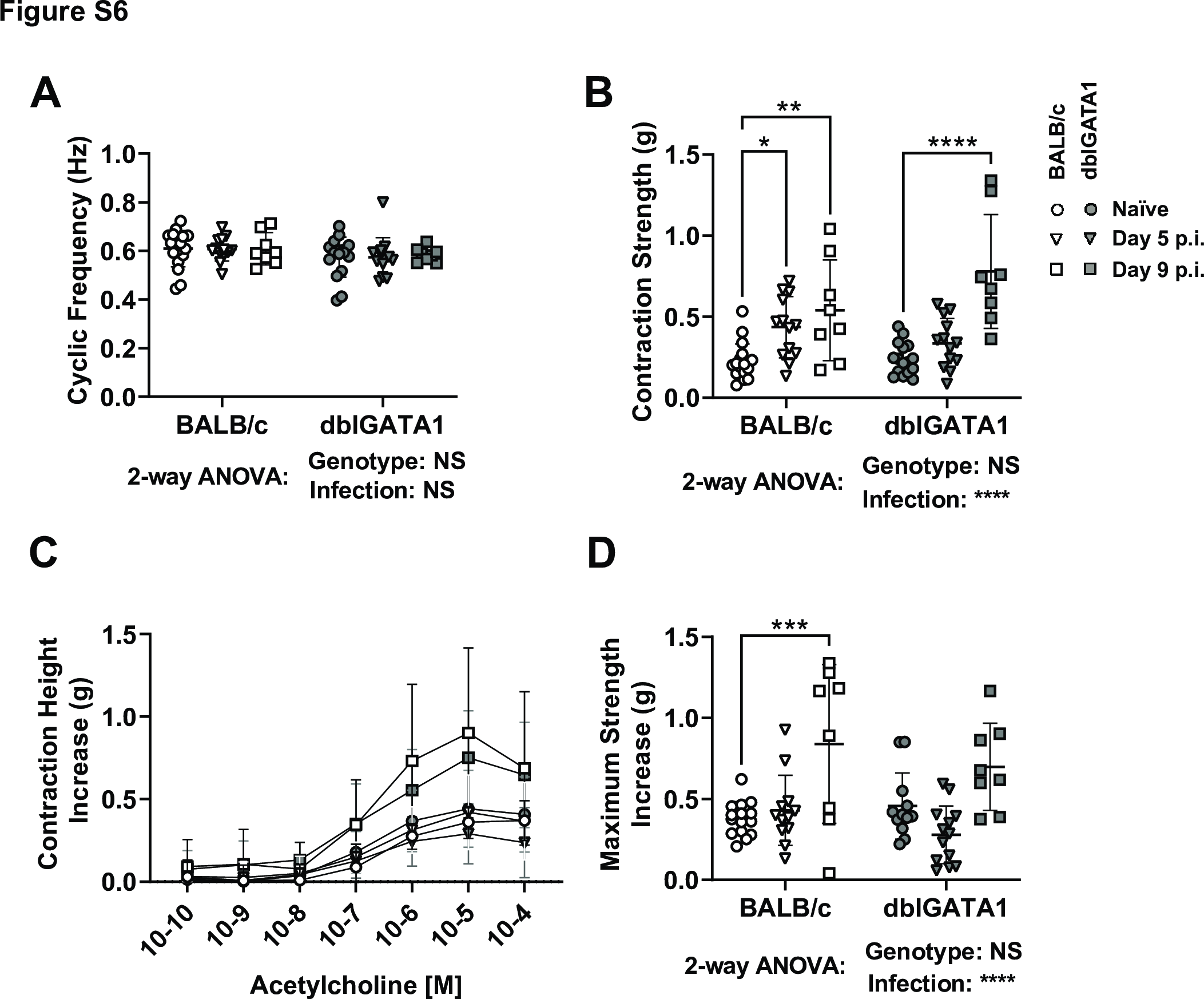

Supplement: S6 Fig — dblGATA1 mice and wildtype BALB/c controls were infected with 400–500 L3 Nb and sacrificed at the indicated days p.i. Control groups of naïve mice were included. Ileal longitudinal muscle segments were mounted longitudinally within an organ bath as described in the Materials and Methods and (A) contraction frequency and (B) contraction strength recorded under baseline conditions. Thereafter the same tissues were subject to stimulation using incremental doses of acetylcholine and (C) the dose-dependent change in contraction strength or (D) the maximum increase in contraction strength determined. Symbols represent individual animals pooled from 2 independent experiments (n = 8–15 animals/group). Data are shown mean ± SEM and significance determined using a two-way ANOVA with Tukey’s post-hoc analysis. (TIF) [file ppat.1011766.s006.tif]

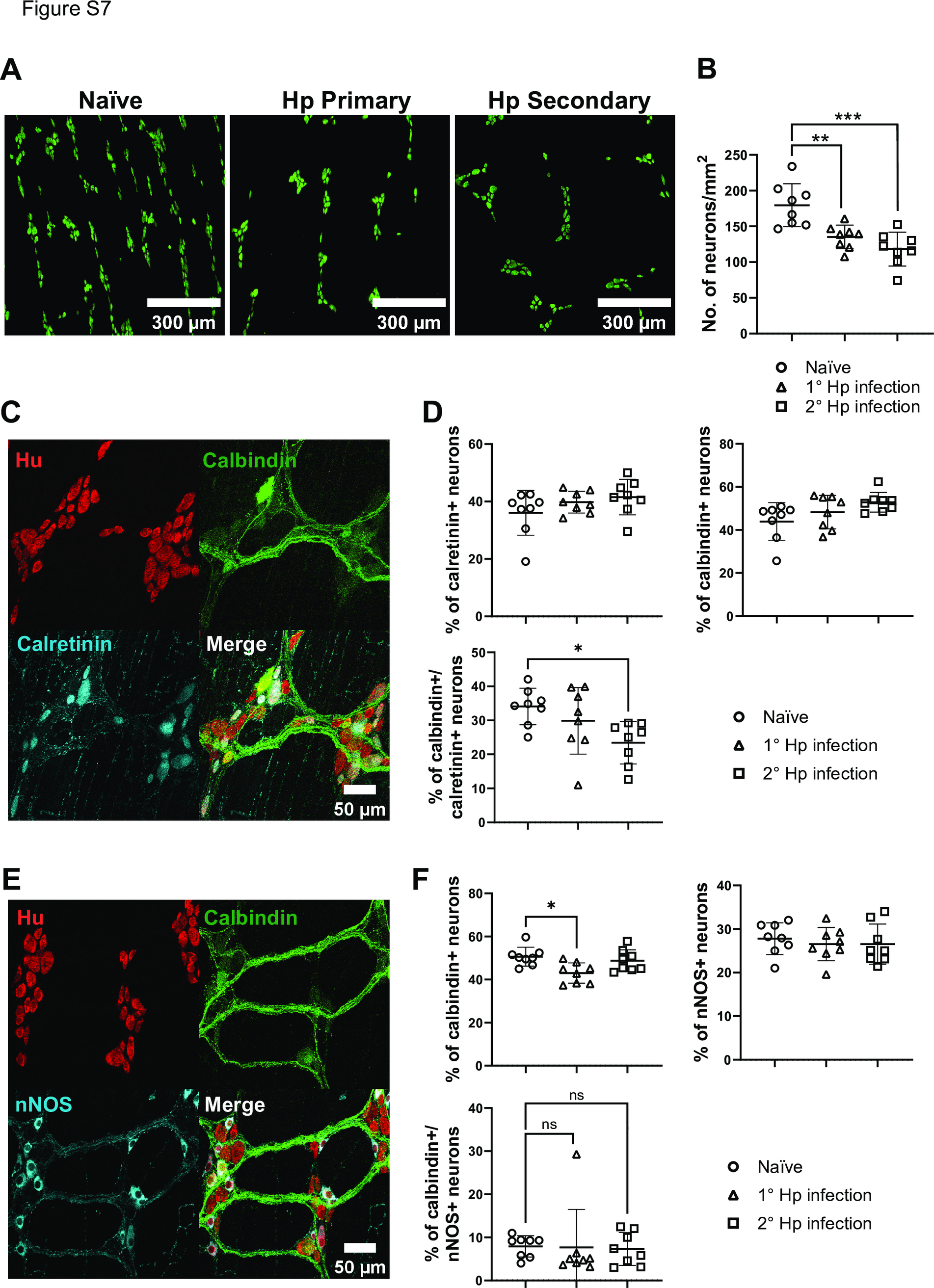

Supplement: S7 Fig — BALB/c mice were infected with 200 L3 Hp at day 0 for the primary infection group or day 0 and day 37 for the secondary infection group and were sacrificed day 14 p.i. or day 51 p.i., respectively. Control groups of naïve mice were included. (A) Representative images of myenteric plexus stained with Hu from a single naïve, primary Hp infected, and secondary Hp infected BALB/c mice. (B) Myenteric neuronal cell density was calculated by counting cell bodies and presented as number/mm2. (C&E) Representative images are shown from a single naïve BALB /c animal for myenteric plexus stained with antibodies directed against C) Hu (red), CalB (green), and CalR (cyan) or E) Hu (red), CalB (green), and nNOS (cyan). (D &F) The percentage of total neurons expressing the indicated neurochemical markers was calculated by counting at least 180 Hu+ cell bodies. Symbols represent individual animals with data pooled from 2 separate experiments with n = 8 animals per group. All data are shown as mean ± SEM and significance determined using a one-way ANOVA with Tukey’s post-hoc analysis. (TIF) [file ppat.1011766.s007.tif]
