# Supplementary material for: Helminth infection driven gastrointestinal hypermotility is independent of eosinophils and mediated by alterations in smooth muscle instead of enteric neurons
Source: PLoS Pathog. 2024 Aug 14;20(8):e1011766. doi: 10.1371/journal.ppat.1011766 (PMC11346963; doi:10.1371/journal.ppat.1011766)
Supplement: Attachment — Submitted filename: Response to reviewer.docx [file ppat.1011766.s008.docx]

Response to reviewers

**Reviewer 1**

Major issues:

1. **It is difficult to know how representative these results are for helminth infections, ie are they specific to acute Nippo infections, or would similar results be seen in any other helminth models? It would be interesting to see if similar mechanisms operate in models such as secondary H. poly infection-**

Author response: We thank the reviewer for the valuable feedback and agree that it would be of high interest to utilise another helminth model. In line with their suggestion we have now utilised the chronic helminth model *H. polygyrus* (Hp) and investigated both primary and secondary infection. As experimental outputs we included intestinal transit, worm counts, smooth muscle thickness, and myenteric neuronal anatomy and neurochemistry and the data is included in a new figure (Line 340-371, Figure 7, and Figure S7). As expected secondary infection was associated with reduced worm burdens, demonstrating protective immunity. Our data also provided more support for a casual role of intestinal transit in worm expulsion as increased transit was observed at day 14 after secondary but not primary infection. Like Nippo infection, Hp infection caused strong alterations to the muscular thickness. Some alterations to the ENS were noted, most notable reduced myenteric neuronal density. However, only very minor changes were observed for neurochemistry. Thus Hp, like Nippo, results in strong myogenic and modest neurogenic alterations.

2. **The main positive finding of the study is that Nippo infection increases smooth muscle contractillity, but that has been well reported in the literature and the cytokines that drive this response have been well characterised (IL-4/13), which the authors acknowledge. Can the authors elaborate on what information the present study has added in this area? Or, is the main area of novelty the fact that neurons and eosinophils are NOT involved much?**

Author response: We agree that the current manuscript replicates a number of known responses, such as the strong type2 dependent alterations to smooth muscle. The main novelty is that it these changes, and not alterations to neuronal signalling, that are responsible for worm expulsion. As the reviewer indicates, the other area of novelty is the that eosinophils are not involved in this process. In addition to this we demonstrate that worm expulsion is tightly linked to increased intestinal transit. This has long been assumed and referred to as being part of the ‘weep and sweep’ response. However, to our knowledge it has never been shown experimentally. Our revised manuscript now also extends the findings to *H. polygyrus* and shows that increased intestinal transit and smooth muscle hypertrophy, but only minor neuronal alterations, are associated with worm expulsion. The lack of involvement of the ENS in helminth expulsion is unexpected and we believe these findings are both important and of high interest for parasite immunologists.

Minor issues:

1. **Lines 123: I would consider adding data from Fig S1 to the main figure set, particularly because the authors have admitted it was "interesting".**

Author response: We thank the review of this suggestion. We have now merged Fig S1 with Fig 2 to have a clear illustration of villous changes in the jejunum and ileum.

**Reviewer 2**

Major issues:

**Nippostrongylus brasiliensis is expelled quickly from the murine intestine, whereas most endemic parasitic helminths chronically reside in the small intestine in infected humans and other mammals. While Nippo infection is an important model used to study the intestinal physiology associated with worm expulsion, expulsion is not the norm. How does chronic infection with Heligmosomoides polygyrus influence intestinal ENS responses and smooth muscle function? It seems beyond the scope of the current report to investigate the role of eosinophils in these responses in H. polygyrus infection, but investigation of how acute vs. chronic infection affects smooth muscle responses to infection is important in establishing the physiological relevance of the data presented.**

Author response: We thank the reviewer for the valuable feedback and agree that it would be of high interest to utilise the chronic helminth model *H. polygyrus* (Hp). In line with the reviewers point (and that of reviewer one) we have now investigated both primary and secondary Hp infection. As experimental outputs we included intestinal transit, worm counts, smooth muscle thickness, and myenteric neuronal anatomy and neurochemistry and the data is included in a new figure (Line 340-371, Figure 7, and Figure S7). As expected secondary infection was associated with reduced worm burdens, demonstrating protective immunity. Our data also provided more support for a casual role of intestinal transit in worm expulsion as increased transit was observed at day 14 after secondary but not primary infection. Like Nippo infection, Hp infection caused strong alterations to the muscular thickness. Some alterations to the ENS were noted, most notable reduced myenteric neuronal density. However, only very minor changes were observed for neurochemistry. Thus Hp, like Nippo, results in strong myogenic and modest neurogenic alterations.

1. **Lines 123: The coding for the days and genotypes is quite complex, as the reader needs to apply the single “legend” across all 4 panels, and the 4 panels have data from different days post-infection. Is there a way to simplify?**

Author response: We thank the review the feedback. We agree that the legend for Figure 1 is complex and as such we brain-stormed about alternative approaches and consulted with all of the co-authors on this matter. In the end we believed that our attempts to simplify the data led to the loss of important statistical information, and given the statistical tests used are most appropriate to the dataset we could not further reduce the complexity of the legend without impairing the scientific integrity of the work. Thus, we kept the same statisical format and methods but instead of annotating significant differences between each individual variable we altered all the graphs to only show significant between each individual variable and the naïve group. This has been explained in greater detail in Materials and Methods and we believe the graphs are now more straightforward to understand.

2. **The stats indications on the bottom of the panels (genotype vs. infection) are confusing in conjunction with the stats already indicated on the graphs.**

Author response: We thank the review this comment. Whilst we agree that the stats at bottom of each panel can be confusing, they are the most appropriate for the dataset and necessary to fully understand the results. Most of our experiments contain two independent groups (genotype vs. infection status) and were analysed using two-way ANOVA. The significance indicated inside the graphs are only between each individual variable and the naive group, which do not represent the overall effect of genotype or infection status. For example, in Figure 3B Nb infection reduced neuronal density when considering both BALB/c and dblGATA mice. However, such a difference was not represented in any of individual groups. This is the standard method to present such data and very powerful in terms of making robust conclusions. Thus we have opted to keep the presentation the same but added more explanation for the reader to the Materials and Methods section (Line 662-672).

**Reviewer 3**

Minor issues:

1. **Much of the paper is about the physiology and physiological changes in the enteric nervous system (ENS) and the myenteric and submucosal plexus. This is introduced in lines 56-68 but it comes back later in the results and discussion. I think a diagram or illustration of these tissues and their relation to lamina propria etc would be informative for the reader and would facilitate understanding of the data. I suggest that the authors add this as a new figure, or perhaps as a panel on existing figure 1.**

Author response: We would like to thank the review for this suggestion. We have now added an illustration of the structure and anatomy of the ENS to Figure 3 as panel A.

2. **Line 169: It’s a bit confusing as currently written. I suggest changing to “Ahrends and Aydin et al., reported that…**

Author response: We have changed this accordingly.
